# Practical Knowledge

**Published:** 1877-02

**Authors:** 


### PRACTICAL KNOWLEDGE.

THE world little imagines how largely it is indebted to the laborious researches of scientific medical men for many of the most important truths relative to human health, happiness and life. As population increases, and the value of food is enhanced, the knowledge which Chemistry has elicited is becoming more and more valuable in a practical point of view.

"How much ability to labor can I derive from eating a pound of potatoes, or a dollar's worth of brandy, beer or gin?" are items which could be turned to large account by multitudes of the toiling poor.

Some kinds of food are more nutritious than others, and if it should be found that articles which are cheapest, have most nutriment, and give the highest ability to labor, then knowledge becomes money to the poor. Tables vary, but some of the general results are as follows: one pound of rice, prepared for the table, gives eighty-eight per cent. of nutriment, and consequently, a relatively proportional ability to labor, compared with other articles of food. A pound of beef, costing fifteen cents, gives only twenty-six per cent. of nutriment. According to these estimates, therefore, rice as an article of food, is one hundred per cent. cheaper, one hundred per cent. more valuable to the common laborer than roast beef, yet countless numbers of the poor in New York, strain a point daily to purchase beef at fifteen cents a pound, when they could get a pound of rice for one-third the amount, the rice too, having three times as much nutriment as the beef, making a practical difference of six hundred per cent., aside from the fact, that boiled rice is three times easier of digestion than roast beef, the rice being digested in about one hour, roast beef requiring three hours and a half. There is meaning, then, in the reputed fact, that two-fifths of the human family live mainly on rice. We compile, therefore, the following tables for preservation, as being practically and permanently useful. All the economist requires, is to compare the price of a pound of food, with the amount of nutriment which it affords.

| Kind of Food. | Mode of Preparation. | Per centage of Nutriment. |
|---|---|---|
| Oils, | raw, | 95. |
| Peas, | boiled | 93. |
| Barley, | boiled | 92. |
| Corn Bread, | baked | 91. |
| Wheat Bread, | baked | 90. |
| Rice, | boiled | 88. |
| Beans, | boiled | 87. |
| Rye Bread, | baked | 79. |
| Oat Meal, | porridge | 74. |
| Mutton, | broiled | 30. |
| Plums, | raw | 29. |
| Grapes, | raw | 27. |
| Beef, | raw | 26. |
| Poultry | roast | 26. |
| Pork, | roast | 24. |
| Veal, | fried | 24. |
| Venison | broiled | 22. |
| Cod Fish, | boiled | 21. |
| Eggs, | whipped | 13. |
| Apples, | raw | 10. |
| Milk, | raw | 7. |
| Turnips, | boiled | 4. |
| Melons, | raw | 3. |
| Cucumbers, | raw | 2. |

---

A Beautiful Compliment to the Physician.—I dare not place any gift, however beautiful, or any success however brilliant, above the talent or the skill which can relieve a single pang, and the self-devotion which lays them at the feet of the humblest fellow creature.—*Oliver Wendell Holmes.*

